# Editorial: Herbal medicines for gastrointestinal and hepatic diseases - novel pharmacological and toxicological approaches, Volume I

**DOI:** 10.3389/fphar.2023.1157229

**Published:** 2023-07-07

**Authors:** Muhammad Hasnat, Mirza Muhammad Faran Ashraf Baig, Mohammad Saleem, Aftab Ullah, Muhammad Faisal Nadeem, Alessandra Durazzo, Massimo Lucarini

**Affiliations:** ^1^ Institute of Pharmaceutical Sciences, University of Veterinary and Animal Sciences, Lahore, Pakistan; ^2^ The Hong Kong University of Science and Technology, Hong Kong, Hong Kong SAR, China; ^3^ University of the Punjab Lahore, Lahore, Pakistan; ^4^ Jiangsu University, Zhenjiang, China; ^5^ University of Veterinary and Animal Sciences, Lahore, Pakistan; ^6^ CREA-Research Centre for Food and Nutrition, Rome, Italy

**Keywords:** herbal medicines, traditional medicines, active ingredients from herbal medicines, GIT, liver, toxicology

After respiratory tract diseases, acute gastrointestinal infections are the second most common infections among infants and children and are responsible for morbidity and mortality (Ferguson et al., 2020). These infections are caused by a variety of microorganisms with the most common species are *Helicobacter pylori, Salmonella species, Clostridium difficile, Shigella species, Giardia lamblia and Escherichia coli* (Shariati et al., 2019). Gastrointestinal system is also associated with the hepatic complications including NAFLD and gastrointestinal malignancies, i.e., HCC (Younossi et al., 2018). Worldwide, liver cancer causes second most cancer related deaths (Jemal et al., 2011). For the management of hepatic and gastrointestinal diseases, long-term strategies are required from government and international bodies because hepatitis B virus and hepatitis C virus infected subjects are 370 million and 130 million, respectively (Alter, 2006). Traditional and complementary medicines (TCMs) are clinical practices that are used in the diagnosis, treatment and prevention of diseases. They are not completely merged into the healthcare system, however they are affordable, accessible and culturally accepted by the people. Herbal products are one of the major part of TCM. It is reported that market share of natural preparations is up to several billions of dollars in developing and developed nations, showing the trust of people on these products (Hitl et al., 2019). Herbal medicines treat gastrointestinal diseases by affecting intestinal barrier, microbial composition and metabolites, and inflammation (Wang et al., 2023).

In the Research Topic of papers under the Research Topic “Herbal Medicines for Gastrointestinal and Hepatic Diseases - Novel Pharmacological and Toxicological approaches - Volume I″, 15 papers were published., mainly focusing the herbal medicines for gastrointestinal and hepatic diseases.

This Research Topic includes the following dimensions: Pre-clinical and clinical studies of herbal products and their biologically active metabolites in the management of GIT and liver diseases. Novel *in vitro* assays for the identification of potentially biologically active compounds for the treatment of gastric and hepatic cancer. Novel cellular and molecular mechanisms that describe the therapeutic effects as well as toxic effects of herbal medicines and their active metabolites on the GIT and liver. The role of the GI-microbiome in preventing and treating gastrointestinal and hepatic diseases.

The papers within this Research Topic carry out interesting themes in the area of research. As instance, Lee et al. found that treatment with Chunggan syrup (CGX) reduced tumor nodules in normal and HFD fed mice. Molecular biology studies explained that CGX antitumor effect was associated with the activation of E- Cadherin and reduction in the expression of VE- Cadherin in liver under MC38 free condition. CGX also reduced liver steatosis *via* modulating AMPk and PPARα. Yuan et al. showed that triptolide (TP), one of the fat-soluble components extracted from the Chinese medicinal herb *Tripterygium wilfordii* Hook F. (TWHF), induced cell death in TNFα-pretreated MKN45 cells and AGS cells. Both TP and TNFα, in combination promoted the gastric cancer cell death through influencing H19/miR-204-5p/NF-κB/FLIP axis.

Ivyna de Araújo Rêgo et al. reported the role of flavonoids in the prevention of gastric cancer by treating *H. pylori* infection and explored that flavonoid rich extracts had anti-*H. pylori* action by inhibiting urease, distortion of genetic material, decreasing protein synthesis and adhesion of microorganism to host cells. Tan et al. published a systematic review and meta-analysis on Chinese herbal medicine with oxaliplatin and showed that these agents improve the tumor response in advance gastric cancer. Brockmueller et al. showed for the first time that β1 integrin partly suppressed the inhibitory effects of resveratrol on the metastasis of colorectal cancer (CRC) cells. Resveratrol inhibited TME induced phosphorylation and nuclear shift of NF- κB which is associated with CXCR-4, MMP-9, FAK and caspase-3. However, β1 integrin inhibited the anti-invasive and anti-metastatic effects of resveratrol.

Several studies have focused the autophagy pathway like, Zhang et al. expressed that Qingluo Tongbi Formula (QTF), a Chinese herbal formula, reduced TWHF-induced hepatotoxicity. TWHF caused an increase in endoplasmic reticulum stress (ERS) that regulated the mitochondrial autophagy through PERK-ATF4 pathway. QTF reduced the TW-induced ERS and mitophagy. Wang et al. concluded that chrysophanol-8-O-glucoside (CPOG) protected mice from LPS/D-galactosamine-induced acute liver damage by decreasing inflammation, oxidative stress and autophagy. CPOG inhibited the levels of p-IκB, p-p65, TNF-α and IL-1β upregulated by LPS. CPOG also reduced the levels of LC3B, P62, ATG5 and Beclin 1 by reducing reactive oxygen species and MAPK pathway. Cheng et al. reported that Huangkui lianchang decoction (HLD), a Chinese herbal preparation, treated colitis by blocking NF-κB pathway and autophagy markers. HLD showed anti-inflammatory effects by increasing the level of IL-10 and decreasing the level of pro-inflammatory cytokines. HLD also reduced the levels of LC3II/I and Beclin 1.

Couple of studies reported ethanol-induced gastric complications, i.e., Ke et al. first time reported that LDOP-1 had protective effect against ethanol-induced gastric mucosal damage by controlling AMPK/mTOR pathway. Xie et al. performed a study on ethanol-induced gastric lesions and showed that MLG could enhance defensive mechanism through NF-κB/Nrf2/HO-1 pathway and had protective effect against Ethanol-induced gastric lesions. This study reported that MLG increased the levels of antioxidant enzymes and reduced the levels of TNF-α and IL-1β. MLG increased the expressions of Nrf-2 and HO-1 and decreased the expressions of COX-2 and NF-κB.

Other studies on herbal medicines treating gastrointestinal and liver diseases have also significant findings. Chen et al. published a clinical evidence base study and expressed that SLBZP had capacity to treat ulcerative colitis. Kang et al. reported the randomized, double-blind, placebo-controlled, multi-center clinical trial study for the assessment of the efficacy and safety of traditional Chinese medicine external washing for treating the postoperative wounds in diabetic patients with anal fistula. Yao et al. proposed the general guidelines about the use of herbal preparations in the treatment of gastrointestinal and liver complications. Sheng et al. showed that lithocholic acid had significant protective activities on the intestinal environment, including the maintenance of tight junctions, anti-bacterial and anti-inflammatory responses. Gao et al. showed that epimedin B caused the Epimedii Folium (EF)-induced Idiosyncratic drug-induced liver injury (IDILI) and the glycogen concentration in EF is not dependent on NLRP3 inflammasome activity. They studied that epimedin B increased the secretion of IL-1β, played a role in the maturation of caspase-1 and activated the NLRP3 inflammasome by increasing the level of ROS.

In conclusion, this Research Topic provided recent advances in the scientific knowledge on the gastrointestinal system.
